# PD‐1 inhibition with retifanlimab and/or arginase inhibition with INCB001158 in Japanese patients with solid tumors: A phase I study

**DOI:** 10.1002/cam4.6980

**Published:** 2024-04-23

**Authors:** Yasutoshi Kuboki, Takafumi Koyama, Nobuaki Matsubara, Yoichi Naito, Shunsuke Kondo, Kenichi Harano, Kan Yonemori, Kiyotaka Yoh, Yuan Gu, Tetsuya Mita, Xuejun Chen, Eiji Ueda, Noboru Yamamoto, Toshihiko Doi, Toshio Shimizu

**Affiliations:** ^1^ Department of Experimental Therapeutics National Cancer Center Hospital East Kashiwa Japan; ^2^ Department of Experimental Therapeutics National Cancer Center Hospital Tokyo Japan; ^3^ Department of Breast and Medical Oncology National Cancer Center Hospital East Kashiwa Japan; ^4^ Department of General Internal Medicine National Cancer Center Hospital East Kashiwa Japan; ^5^ Department of Thoracic Oncology National Cancer Center Hospital East Kashiwa Japan; ^6^ Incyte Corporation Wilmington Delaware USA; ^7^ Incyte Biosciences Japan G.K. Tokyo Japan; ^8^ Department of Medical Oncology/Cancer Center Wakayama Medical University Hospital, Wakayama Medical University Graduate School of Medicine Wakayama Japan

**Keywords:** arginase, Japan, PD‐1 inhibitor, retifanlimab, solid tumors

## Abstract

**Background:**

Retifanlimab is a humanized monoclonal antibody targeting programmed death protein‐1, and INCB001158 is an oral arginase inhibitor. This phase Ib study investigated retifanlimab, INCB001158, and their combination in Japanese patients with advanced solid tumors.

**Methods:**

Patients received retifanlimab (500 mg every 4 weeks [Q4W] i.v.) or escalating doses of INCB001158 (75 or 100 mg twice daily [BID]) monotherapy in Part 1 and combination of retifanlimab (500 mg Q4W) and INCB001158 (100 mg BID) in Part 2. Primary endpoints were safety, tolerability, dose‐limiting toxicities (DLTs), and determination of recommended phase II doses in Japanese patients.

**Results:**

Eighteen patients (retifanlimab or INCB001158 monotherapy and combination; *n* = 6 each) were enrolled at 2 sites in Japan. There were no DLTs, fatal adverse events (AEs), or discontinuations due to AEs. Rash (all grade 1) was the most common treatment‐emergent AE with retifanlimab (*n* = 6). Treatment‐related AEs were reported with retifanlimab (*n* = 4) or INCB001158 (*n* = 2) monotherapy and with combination (*n* = 4); an immune‐related AE (thyroid disorder, grade 2) was reported with combination. Two responses were observed with retifanlimab monotherapy (1 complete, 1 partial) and 1 stable disease (SD), for an overall response rate of 33.3% (95% confidence interval [CI], 4.3–77.7) and disease control rate (DCR) of 50% (95% CI, 11.8–88.2). Three patients had SD with INCB001158 monotherapy (DCR 50%; 95% CI, 11.8–88.2). No responses or SD were observed with combination therapy.

**Conclusion:**

Retifanlimab, INCB001158, and their combination had acceptable safety profiles. Promising retifanlimab antitumor activity warrants further investigation in Japanese patients.

## Graphical abstract

POD1UM‐104 evaluated the safety, tolerability, dose‐limiting toxicities (DLTs), and recommended phase II dose (RP2D) of retifanlimab (PD‐1 inhibitor, A), INCB001158 (arginase inhibitor, B), and their combination (retifanlimab + INCB001158, C) in Japanese patients with advanced solid tumors. There were no DLTs and no adverse events with fatal outcomes or leading to treatment discontinuation reported; the overall response rate was 33.3% and disease control rate was 50.0% with retifanlimab monotherapy. At the RP2D of 500 mg administered every 4 weeks, retifanlimab had a manageable safety profile and demonstrated encouraging antitumor activity, supporting further evaluation in Japanese patients.

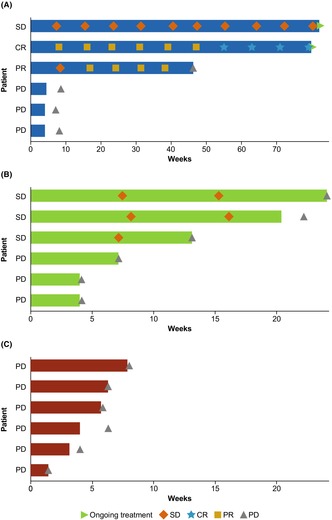

## 1 INTRODUCTION

Inhibitors of programmed death protein‐1 (PD‐1), or its ligand, programmed death ligand‐1 (PD‐L1), have become established immunotherapies for the treatment of patients with a diverse range of cancers.^1^, ^2^, ^3^ Retifanlimab (INCMGA00012) is a humanized, hinge‐stabilized immunoglobulin G4κ monoclonal antibody targeting PD‐1 with preclinical characteristics similar to the immune checkpoint blockade (ICB) class.^4^ Retifanlimab had a manageable safety profile and demonstrated promising early signs of antitumor activity in a phase I study in patients with advanced solid tumors,^5^, ^6^ including in a cohort of patients with microsatellite instability‐high (MSI‐H) or mismatch repair deficient (dMMR) endometrial cancer^7^ (which accounts for ~25% of endometrial cancers^8^). Retifanlimab also showed encouraging efficacy in phase II studies, one in patients with advanced or metastatic squamous carcinoma of the anal canal (SCAC)^9^ and another in Merkel cell carcinoma.^10^ Other phase II studies of retifanlimab are ongoing, and phase III studies in combination with platinum‐based chemotherapy are currently recruiting in patients with chemotherapy‐naive SCAC and non‐small cell lung cancer.

Response rates following anti‐PD‐(L)1 monotherapy vary widely depending on the type of malignancy being treated.^2^, ^3^ A significant proportion of patients are refractory to ICB, and among those who do respond, many develop resistance.^2^ Reasons may include concurrent immune inhibitory mechanisms operative in the tumor microenvironment or activation of additional escape mechanisms to evade ICB by PD‐(L)1 inhibitors.^2^, ^11^ There is a clear unmet need to deepen and/or lengthen responses and overcome resistance to PD‐(L)1 inhibitors; therefore, targeting one of the concurrent immune inhibitory mechanisms using treatment combinations is an attractive approach.^11^, ^12^

The enzyme arginase is expressed by tumor‐infiltrating myeloid cells, including tumor‐associated macrophages, myeloid‐derived suppressor cells, and granulocytes, and depletes extracellular levels of the amino acid arginine in the tumor microenvironment.^2^, ^13^ T cells require arginine for rapid, successive rounds of replication following T‐cell antigen receptor activation. Thus, arginase can contribute to immunosuppression; conversely, inhibition of arginase could restore arginine metabolism in favor of lymphocyte proliferation, with antitumor effects.^13^

INCB001158 is an oral, potent, and selective inhibitor of arginase.^13^ In preclinical studies, INCB001158 restored T‐cell proliferation in the presence of inhibitory granulocytes, demonstrated antitumor activity in mouse models, and synergistically inhibited tumor growth when combined with ICB.^13^ In phase I/II studies, INCB001158 was well tolerated,^14^, ^15^ and was shown to inhibit arginase, leading to an increase in plasma arginine levels.^14^ INCB001158 demonstrated preliminary clinical activity both as monotherapy and in combination with pembrolizumab and gemcitabine‐cisplatin chemotherapy.^14^, ^15^

Previous studies have shown no difference in the pharmacokinetics (PK) and safety of the anti‐PD‐1 agents pembrolizumab and nivolumab in Japanese patients compared with Western patients.^16^, ^17^ To date, there are no published reports on the combination of retifanlimab (PD‐1 inhibitor) and INCB001158 (arginase inhibitor). Therefore, this study was conducted to assess the safety, tolerability, PK, and recommended phase II dose (RP2D) of retifanlimab, INCB001158, and their combination in Japanese patients with advanced solid tumors.

## 2 METHODS

### 2.1 Study design

This was a phase Ib, open‐label, multicenter study (POD1UM‐104 [NCT03910530]). The study was conducted in 2 parts. In Part 1, patients received single‐agent retifanlimab or escalating doses of single‐agent INCB001158 to confirm the RP2D of each agent. Single‐agent dosing was performed to comply with local regulations. In Part 2, patients received the combination of retifanlimab and INCB001158 at their respective RP2D determined in Part 1, to further explore safety and preliminary evidence of clinical activity. Treatment cycles in Part 1 and Part 2 were 28 days, and patients could receive treatment for up to 2 years, or until disease progression, unacceptable toxicity, death, or patient withdrawal.

In Part 1, 6 patients received retifanlimab 500 mg every 4 weeks (Q4W), given as an i.v. infusion over 60 min; this dose was based on the RP2D determined from global studies. The starting dose of INCB001158 was 75 mg taken orally twice daily (BID). Dose escalation of INCB001158 followed a standard 3 + 3 design; if 75 mg BID was deemed to be tolerable in at least 3 patients, a 100‐mg BID dose level cohort comprising at least 3 patients would be opened.

In Part 2, if 1 or fewer dose‐limiting toxicities (DLTs) were observed in the first 6 patients, the retifanlimab and INCB001158 dose level would be considered tolerable. DLTs were assessed by the investigator according to the National Cancer Institute (NCI) Common Terminology Criteria for Adverse Events (CTCAE) v5.0 criteria (Table S1).

### 2.2 Study population

Patients were Japanese (born in Japan, not lived outside of Japan for over 10 years, and able to trace maternal and paternal Japanese ancestry) adult men and women at least 18 years of age with histologically or cytologically confirmed diagnosis of any locally advanced or metastatic solid tumor not amendable to local or other curative therapy. Patients had an ECOG performance status of 0 or 1 and a life expectancy of >3 months.

Exclusion criteria included anticancer therapy or radiotherapy within 14 days, or immunotherapy or biological therapy within 21 days, of first dose of study treatment; prior systemic treatment with an arginase inhibitor; immune‐related toxicity during prior ICB therapy; active autoimmune disease requiring immunosuppression; and known central nervous system metastases and/or carcinomatous meningitis.

### 2.3 Study endpoints and assessments

The primary endpoints of the study were safety, tolerability, DLTs, and RP2D of single‐agent retifanlimab, INCB001158, and the combination. Adverse events (AEs) were graded by NCI CTCAE v5.0, and immune‐related adverse events (irAEs) were monitored as AEs of special interest. Physical examinations, monitoring of vital signs, electrocardiograms, and laboratory assessments were conducted throughout the study.

Pharmacokinetics of single‐agent retifanlimab and INCB001158, and the combination, were secondary endpoints of the study. The detection of anti‐drug antibodies (ADAs) was an exploratory endpoint. Blood samples for retifanlimab PK assessments were taken on days 1 (−2 h pre‐dose, end of infusion, and 6 h post‐dose), 2, 8, and 15 (no specified time) of cycle 1, day 1 of cycles 2–8 (−2 h pre‐dose, and end of infusion on cycles 2 and 6 only), and day 1 of every 4 cycles after cycle 8 (−2 h pre‐dose). Samples for ADA assessment were collected at the same time as for PK analysis, and an additional sample was collected at safety follow‐up on day 15 (no specified time). Blood samples for INCB001158 were collected pre‐dose, 0.5, 1, 2, 4, 6, and 8 h post‐dose on days 1 and 15 of cycle 1, 12 h post‐dose on day 1 of cycle 1, and pre‐dose on days 2 and 8 for cycle 1, and day 1 of cycle 2. Serum concentrations of retifanlimab were determined using an electrochemiluminescence immunoassay method, and plasma concentrations of INCB001158 were measured using a validated liquid chromatography with tandem mass spectrometry assay (Incyte Corporation, Wilmington, DE, USA). ADAs against retifanlimab were monitored using a bridging immunoassay (Incyte Corporation).

Pharmacokinetics parameters were derived from concentrations versus time data using model‐independent noncompartmental analysis methods (WinNonlin v8.2, Certara USA Inc., Princeton, NJ, USA), and included maximum observed serum or plasma concentration (*C*_max_), serum or plasma concentration observed at the end of the dosing interval (*C*_tau_), time to maximum observed serum or plasma concentration (*t*_max_), area under the serum or plasma concentration–time curve (AUC) from time 0 to the end of the dosing interval (AUC_tau_), AUC from time 0 to infinity (AUC_0‐∞_), apparent terminal‐phase disposition half‐life, clearance, and volume of distribution.

Preliminary clinical activity of single‐agent retifanlimab and INCB001158, and the combination, was an additional secondary endpoint. Diagnostic imaging was performed by computed tomography scan every 8 ± 1 weeks. Clinical activity was assessed by measuring overall response rate (ORR), defined as the percentage of patients experiencing a complete or partial response (CR or PR) as determined by the investigator according to Response Evaluation Criteria in Solid Tumors (RECIST) v1.1; disease control rate (DCR), defined as the percentage of patients maintaining either an objective response or stable disease (SD) for at least 12 weeks per RECIST v1.1; and duration of response (DOR), defined as the time from first observed response until onset of progressive disease (PD) as determined by the investigator according to RECIST v1.1 or death from any cause.

Pharmacodynamics of INCB001158 alone or in combination were assessed as an exploratory objective. Urine was collected at regular intervals from patients in the INCB001158 monotherapy and combination groups to assess urinary orotic acid levels and evaluate whether INCB001158 inhibits the urea cycle. Plasma samples from patients in these groups were also assessed for ammonia, arginine, and ornithine levels.

### 2.4 Statistical analysis

Safety and efficacy analyses were conducted in the full analysis set, which comprised all enrolled patients who received at least 1 dose of the study drug. PK analyses were conducted in the PK population, which included patients who received at least 1 dose of study drug and had a baseline and at least 1 post‐dose PK sample.

Safety data were summarized using descriptive statistics. PK parameters were summarized by study part, treatment regimen, cohort, dose levels, and/or visit. ORR and DCR were reported with their exact 95% confidence intervals (CIs); DOR was based on the Kaplan–Meier estimate of the distribution function, and the estimated median along with 95% CI was reported. Statistical analyses were performed using SAS® software v9.4 or later (SAS Institute Inc., Cary, NC, USA).

Sample sizes were selected to determine safety and tolerability in Japanese patients and to ensure adequate samples were available for PK analysis. Sample sizes were not hypothesis based.

## 3 RESULTS

### 3.1 Patients

Eighteen patients (6 in each group of retifanlimab monotherapy, INCB001158 monotherapy, and combination treatment) were enrolled at sites in Japan between August 6, 2019 and January 30, 2020. The cutoff date for this analysis was March 5, 2021. The median age was 54.5–69.5 years across treatment groups, and patients were predominantly female (78%) (Table 1). The most common tumor types were endometrial (*n* = 4), breast (*n* = 3), colorectal (*n* = 3), and lung (*n* = 2). None of the patients with endometrial cancer were known to have MSI‐H or dMMR tumors. All patients had received prior systemic therapy, 78% had prior surgery, and 39% had prior radiation therapy.

**TABLE 1:** Patient baseline demographics and clinical characteristics (full analysis set).

	Retifanlimab (*n* = 6)	INCB001158 (*n* = 6)	Retifanlimab + INCB001158 (*n* = 6)
Age (years), median (range)	67.0 (46–71)	69.5 (52–75)	54.5 (46–60)
≥65 years, *n* (%)	4 (66.7)	4 (66.7)	0
Sex, *n* (%)			
Male	0	3 (50.0)	1 (16.7)
Female	6 (100.0)	3 (50.0)	5 (83.3)
Weight (kg), median (range)	55.2 (43.9–67.2)	47.6 (40.8–72.1)	58.5 (48.3–71.3)
BMI (kg/m^2^), median (range)	22.3 (16.9–29.5)	19.9 (15.5–26.2)	23.4 (19.6–26.2)
Time since initial diagnosis (years), median (range)	3.3 (1.7–17.4)	2.5 (0.9–4.2)	5.3 (1.6–11.7)
ECOG status, *n* (%)			
0	5 (83.3)	4 (66.7)	3 (50.0)
1	1 (16.7)	2 (33.3)	3 (50.0)
Solid tumor type, *n* (%)			
Endometrial cancer	3 (50.0)	1 (16.7)	0
Breast cancer	1 (16.7)	0	2 (33.3)
Colorectal cancer	1 (16.7)	2 (33.3)	0
Lung cancer	1 (16.7)	0	1 (16.7)
Other	0	3 (50.0)^a^	3 (50.0)^b^
Metastatic disease, *n* (%)	6 (100.0)	6 (100.0)	6 (100.0)
Prior systemic therapy, *n* (%)	6 (100.0)	6 (100.0)	6 (100.0)
Prior surgery, *n* (%)	5 (83.3)	5 (83.3)	4 (66.7)
Prior radiation therapy, *n* (%)	4 (66.7)	1 (16.7)	2 (33.3)

Abbreviation: BMI, body mass index.

^a^ One pancreatic cancer, 1 sarcoma, and 1 cancer of unknown primary origin.

^b^ One ovarian cancer, 1 vaginal cancer, and 1 rectal cancer.

In the retifanlimab monotherapy group, patients received a median of 7 infusions (range, 2–21), with a median dose of 500 mg and a median duration of treatment of 5.9 months (range, 1.0–19.0). In the INCB001158 monotherapy group, 3 patients received 75 mg BID and 3 received 100 mg BID—these patients have been pooled for subsequent analyses. Patients in the INCB001158 monotherapy group received a median daily dose of 173 mg (range, 147–198), with a median duration of treatment of 2.3 months (range, 0.9–5.6). In the combination group, patients received a median of 1.5 retifanlimab 500 mg infusions (range, 1–2), with a median duration of treatment of 0.5 months (0.03–1.3), and a median daily INCB001158 dose of 196 mg (range, 161–198), with a median treatment duration of 1.1 months (range, 0.3–1.8).

At the time of data cutoff, 16 of 18 patients (89%) had discontinued owing to PD; 2 patients with metastatic endometrial cancer in the retifanlimab monotherapy group remained on treatment.

### 3.2 Safety

There were no DLTs or fatal AEs reported in the study (Table 2). Most patients experienced a treatment‐emergent adverse event (TEAE; 94.4%), and 55.6% had a treatment‐related TEAE (TRAE) of any grade. The most common TEAE was rash (grade 1, *n* = 6), occurring in 3 patients (50%) in each of the retifanlimab and combination groups (Table 3). Other TEAEs reported in ≥2 patients overall were constipation (grade 1) in 2 patients in the retifanlimab group and 1 patient in the INCB001158 group; amylase increased (grade 1, *n* = 1; grade 2, *n* = 1); pruritus (*n* = 2; both grade 1) in the retifanlimab group; tumor pain in 3 patients in the INCB001158 group (grade 1, *n* = 2; grade 2, *n* = 1); and pyrexia in 2 patients in the retifanlimab group (both grade 1) and 1 patient in the combination group (grade 2).

**TABLE 2:** Summary of patients experiencing TEAEs (full analysis set).

Patients experiencing adverse events, *n* (%)	Retifanlimab (*n* = 6)	INCB001158 (*n* = 6)	Retifanlimab + INCB001158 (*n* = 6)
TEAE	6 (100.0)	5 (83.3)	6 (100.0)
Serious TEAE	2 (33.3)	1 (16.7)	0
Grade ≥3 TEAE	2 (33.3)	0	0
Fatal TEAE	0	0	0
DLT	0	0	0
Treatment‐related TEAE	4 (66.7)	2 (33.3)	4 (66.7)
Retifanlimab infusion interruption because of a TEAE	1 (16.7)	N/A	0
Dose delay/treatment interruption because of a TEAE	2 (33.3)	2 (33.3)	2 (33.3)^a^
Treatment discontinuation because of a TEAE	0	0	0

Abbreviations: DLT, dose‐limiting toxicity; N/A, not applicable; TEAE, treatment‐emergent adverse event.

^a^ Included retifanlimab dose delay and INCB001158 interruption.

**TABLE 3:** TEAEs occurring in more than 2 patients overall by preferred term (full analysis set).

Preferred term, *n* (%)	Retifanlimab (*n* = 6)	INCB001158 (*n* = 6)	Retifanlimab + INCB001158 (*n* = 6)
Rash	3 (50.0)	0	3 (50.0)
Constipation	2 (33.3)	1 (16.7)	0
Pyrexia	2 (33.3)	0	1 (16.7)
Tumor pain	0	3 (50.0)	0
ALT increased	1 (16.7)	0	1 (16.7)
Amylase increased	2 (33.3)	0	0
Anemia	1 (16.7)	1 (16.7)	0
AST increased	1 (16.7)	0	1 (16.7)
Blood creatinine increased	1 (16.7)	0	1 (16.7)
Diarrhea	1 (16.7)	1 (16.7)	0
Nausea	1 (16.7)	0	1 (16.7)
Pruritus	2 (33.3)	0	0

Abbreviations: ALT, alanine aminotransferase; AST, aspartate aminotransferase; TEAE, treatment‐emergent adverse event.

In the retifanlimab monotherapy group, 4 patients reported TRAEs: infusion‐related reactions (*n* = 1), pruritus (*n* = 1), rash (*n* = 1), and 1 patient who reported multiple TRAEs, consisting of anemia worsening, fatigue, diarrhea, pruritus, rash, elevated lipase, increased alanine aminotransferase (ALT), and increased aspartate aminotransferase (AST). In the INCB001158 monotherapy group, 2 patients reported TRAEs: diarrhea (*n* = 1) and anemia and hyperbilirubinemia (*n* = 1). In the combination group, 4 patients reported TRAEs: rash (considered related to both study drugs [*n* = 2]); thyroid disorder, increased ALT and AST (considered related to retifanlimab; *n* = 1); rash and skin atrophy (considered related to retifanlimab; *n* = 1). One irAE was reported in 1 patient in the combination group, a grade 2 thyroid disorder that was considered related to retifanlimab.

Serious TEAEs included grade 3 femoral neck fracture (*n* = 1), grade 3 ileus and pyelonephritis (*n* = 1) in the retifanlimab monotherapy group, and grade 2 inguinal hernia (*n* = 1) in the INCB001158 monotherapy group, none of which were considered related to study drug. Grade 3 AEs reported (but not occurring during the treatment window) were lymphocyte count decreased (*n* = 1 in the retifanlimab group), anemia (*n* = 1 in the INCB001158 group), and increased gamma‐glutamyl transferase (*n* = 1 in the combination group).

One patient in the retifanlimab group experienced 2 infusion‐related reactions resulting in infusion interruptions. Treatment interruptions due to TEAEs occurred in 2 patients in each of the three treatment groups; interruptions in the combination group included both retifanlimab and INCB001158. No patients discontinued because of a TEAE.

One patient receiving retifanlimab monotherapy experienced a serious TEAE that occurred after data cutoff. The event of myocarditis required hospitalization and occurred on study day 690. Retifanlimab dosing was interrupted, and the myocarditis was considered resolved after standard of care treatment. The serious TEAE was considered a result of viral or bacterial infection (based on presence of a high inflammatory reaction assessed by procalcitonin, white blood count, and C‐reactive protein levels) and not related to retifanlimab, but dosing was not restarted as the patient had reached the maximum treatment period of 2 years.

### 3.3 Pharmacokinetics

Following the first dose of retifanlimab monotherapy in Japanese patients (*n* = 6), the geometric mean *C*_max_ was 209 mg/L, geometric mean *C*_tau_ at the end of the 4‐week dosing period was 33.9 mg/L, median *t*_max_ was 1.9 h, and geometric mean AUC_tau_ and AUC_0‐∞_ were 1910 mg•day/L and 2990 mg•day/L, respectively (Table S2, Figure S1A). The geometric mean half‐life of retifanlimab was 21.2 days (range, 16.1–30.4 days), geometric mean clearance was 0.167 L/day, and volume of distribution was 5.12 L. Retifanlimab trough concentrations generally increased from cycle 1 to cycle 7 in the 3 patients with available data (Figure S1B). Following the first dose of retifanlimab administered in combination with INCB001158 (*n* = 3), retifanlimab geometric mean *C*_max_ was 187 mg/L, geometric mean *C*_tau_ was 19.0 mg/L, median *t*_max_ was 0.82 h, and geometric mean AUC_tau_ was 2280 mg•day/L (Table S3).

Pharmacokinetics data with INCB001158 monotherapy were available for 6 patients total (3 each at 75‐mg BID and 100‐mg BID dose levels). Following first dose at 100 mg BID, INCB001158 geometric mean *C*_max_ was 1740 ng/mL, median *t*_max_ was 3.9 h, geometric mean AUC_tau_ and AUC_0‐∞_ were 13,700 ng•h/mL and 17,900 ng•h/mL, respectively (Table S4, Figure S2A). At steady‐state with the 100‐mg BID dose, INCB001158 geometric mean *C*_max_ was 2690 ng/mL, median *t*_max_ was 3.8 h, geometric mean AUC_tau_ was 23,200 ng•h/mL, and geometric mean *C*_tau_ was 1130 ng/mL. In combination with retifanlimab, the PK of INCB001158 were comparable to INCB001158 monotherapy in Japanese patients (Table S4 and Figure S2B). No retifanlimab ADAs were detected in samples tested from 12 Japanese patients.

In Japanese patients, the geometric mean *C*_max_, AUC_tau_, and AUC_0‐∞_ following first dose were 31%, 47%, and 66% higher, and mean *C*_tau_ at steady state was 43% higher, compared with Western patients in study POD1UM‐101 (NCT03059823; Table S2). Geometric mean clearance and volume of distribution were comparable in the 2 patient populations when normalized for body weight (0.00310 L/day/kg and 0.0955 L/kg, respectively, in Japanese patients [POD1UM‐104]; 0.00405 L/day/kg and 0.0857 L/kg, respectively, in Western patients [POD1UM‐101]).

### 3.4 Clinical activity

In the retifanlimab monotherapy group, there was 1 CR, 1 PR, and 1 SD, giving an ORR of 33.3% (95% CI, 4.3–77.7) and a DCR of 50.0% (95% CI, 11.8–88.2; Table 4). CR occurred in a patient with endometrial cancer and was ongoing at the time of data cutoff, with a treatment duration of 18.4 months and DOR of 16.4 months; PR occurred in a patient with lung cancer, with a treatment duration of 10.7 months and DOR of 6.8 months; and SD occurred in a patient with endometrial cancer who had received treatment for 19.0 months and was ongoing at time of data cutoff.

**TABLE 4:** Best overall response according to RECIST v1.1 by investigator assessment (full analysis set).

	Retifanlimab (*n* = 6)	INCB001158 (*n* = 6)	Retifanlimab + INCB001158 (*n* = 6)
Best overall response, *n* (%)			
Complete response	1 (16.7)	0	0
Partial response	1 (16.7)	0	0
Stable disease	1 (16.7)	3 (50.0)	0
Progressive disease	3 (50.0)	3 (50.0)	6 (100.0)
Objective response rate, *n* (%) [95% CI]	2 (33.3) [4.3–77.7]	0 [0–45.9]	0 [0–45.9]
Disease control rate, *n* (%) [95% CI]	3 (50.0) [11.8–88.2]	3 (50.0) [11.8–88.2]	0 [0–45.9]

Abbreviations: CI, confidence interval; RECIST, Response Evaluation Criteria in Solid Tumors.

There were 3 SDs in the INCB001158 group, giving a DCR of 50.0% (95% CI, 11.8–88.2). The patients with SD had sarcoma, endometrial cancer, and pancreatic cancer; treatment duration was more than 13 weeks. There were no responses or SDs in the combination group.

**FIGURE 1:**
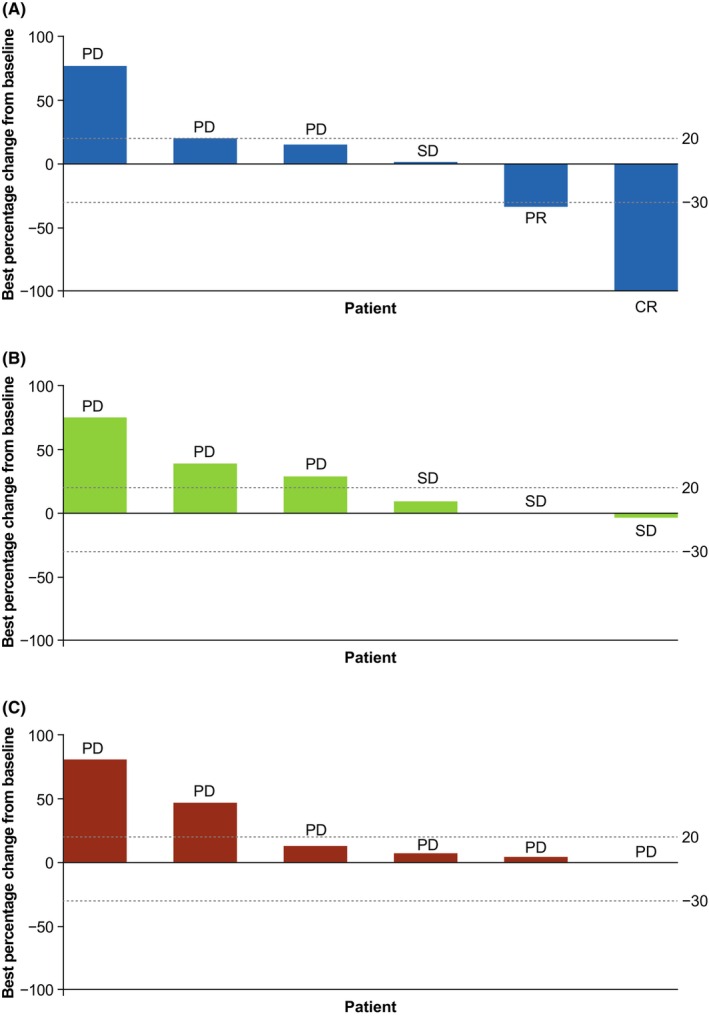
Best percentage change from baseline in target lesion size (full analysis set): (A) retifanlimab, (B) INCB001158, (C) retifanlimab + INCB001158. CR, complete response, PD, progressive disease; PR, partial response; SD, stable disease.

**FIGURE 2:**
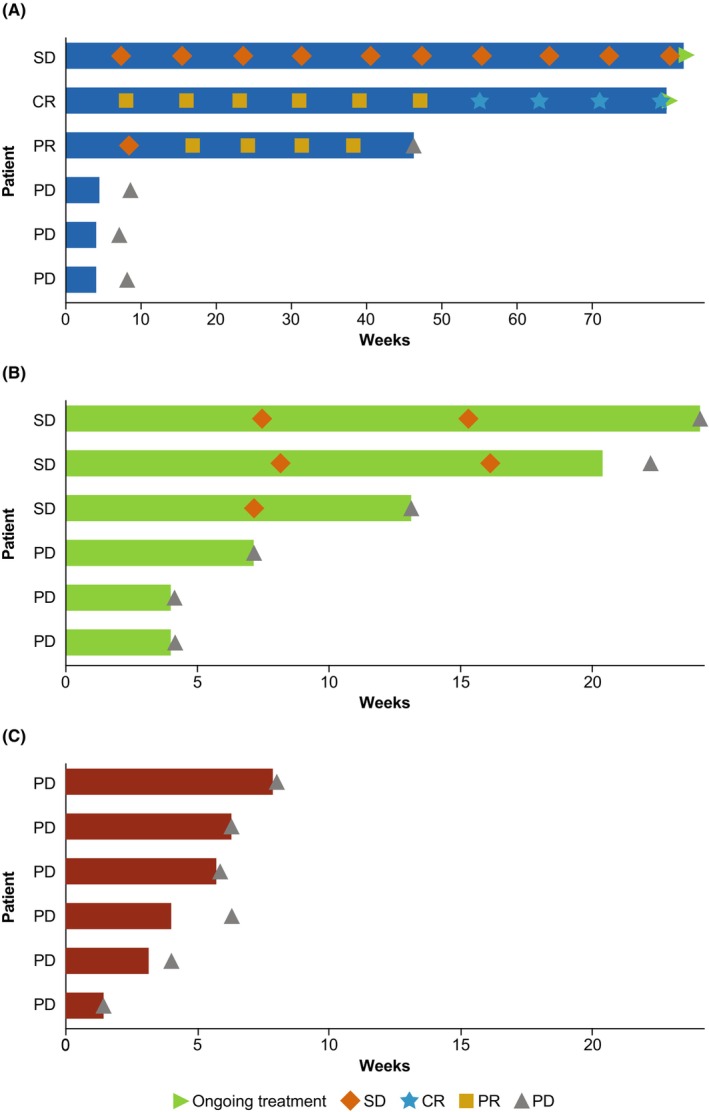
Duration of treatment (full analysis set): (A) retifanlimab, (B) INCB001158, (C) retifanlimab + INCB001158. CR, complete response, PD, progressive disease; PR, partial response; SD, stable disease.

Figure 1 shows the best percentage change from baseline in target lesion size and Figure 2 shows DOR. The median DOR for retifanlimab was not estimable (95% CI, 6.8 months‐not estimable), with a median (range) follow‐up time of 11.6 months (6.8–16.4).

### 3.5 Pharmacodynamics

Immediate and consistent increases in plasma arginine levels in patients receiving INCB001158 monotherapy or in combination with retifanlimab were observed (Figure S3A). There were generally no marked rises in urine orotic acid (Figure S3B), except for increases from baseline observed 6 h post‐dose in 2 patients in the combination group on cycle 1 day 15. There was no evidence of consistent urea cycle inhibition in patients receiving INCB001158, as suggested by no concurrent increases in plasma ammonia (Figure S3C). Additionally, there were no symptoms (e.g., unexplained anorexia, nausea, vomiting) of significant urea cycle inhibition in these patients.

## 4 DISCUSSION

Retifanlimab, INCB001158, and their combination had a manageable safety profile in this phase Ib study in Japanese patients with relapsed or refractory metastatic solid tumors. There were no new or unexpected AEs with retifanlimab, no DLTs or evidence of urea cycle inhibition with INCB001158, and only 1 irAE, which occurred in the combination group. TRAEs were generally manageable with dose delays or interruptions, and no patients discontinued treatment due to a TEAE. These findings are similar to other previously reported studies of retifanlimab,^5^, ^6^, ^9^ and INCB001158.^14^, ^15^ Similar to phase I studies of other anti‐PD‐1 agents (pembrolizumab and nivolumab),^16^, ^17^ our study demonstrated the safety of retifanlimab in Japanese patients at the previously determined RP2D of 500 mg Q4W. Pharmacokinetic analyses indicated that although overall retifanlimab exposures were higher in Japanese patients relative to Western patients, PK parameters were comparable between the 2 populations when normalized for body weight. In addition, PK of retifanlimab and INCB001158 in the combination cohort were comparable to that in monotherapy cohorts, indicating that there is no drug–drug interaction between retifanlimab and INCB001158, which is consistent with the metabolism pathway of both drugs. No ADAs toward retifanlimab were observed in this study, which is consistent with low incident rate of ADAs from other retifanlimab studies (Data on file, Incyte Corporation).

There was preliminary evidence of clinical activity in the retifanlimab monotherapy group, with a CR lasting over 18 months in a patient with endometrial cancer, a PR of 10.7 months' duration in a patient with lung cancer, and SD lasting 19 months in another patient with endometrial cancer. Responses were also observed with retifanlimab in the first‐in‐human study, in which 6 of 26 patients (23%) with non‐small cell lung cancer and 3 of 29 patients (10%) with endometrial cancer had a response^6^; in a follow‐up analysis in the cohort of patients with recurrent MSI‐H or dMMR endometrial cancer, 33 of 76 patients (43%) had an objective response to retifanlimab.^7^ In a phase II study of retifanlimab in patients with SCAC, 14% had a response and another 35% had SD.^9^ Although the low number of patients in the retifanlimab monotherapy group of the present study makes it hard to draw firm conclusions, an ORR of 33% and a DCR of 50% are promising.

No responses were observed in either of the groups receiving INCB001158; however, half of the INCB001158 monotherapy group had SD. It is not known why no disease control was observed in the combination group when there were responses or SDs in the monotherapy groups. The result may be attributable to a relatively low number of patients in each treatment group or the cancer types included. Notably, although the retifanlimab monotherapy group contained 3 patients with endometrial cancer (1 CR and 1 SD), the combination group contained no patients with endometrial cancer; both groups contained 1 patient with lung cancer (1 PR in retifanlimab monotherapy group).

The doses of INCB001158 used were similar to those in other phase I and II studies of INCB001158,^14^, ^15^ and the observed increase in plasma arginine levels suggested that the compound was pharmacologically active in patients in this study. In a previous study including INCB001158 monotherapy, 1 PR and a DCR of 27% were observed in 33 patients with confirmed microsatellite‐stable metastatic colorectal cancer.^14^ Although limited in sample size, our study suggests that INCB001158 may not be significantly active as a monotherapy in other tumor types. INCB001158 has been administered in combination with pembrolizumab, and 3 PRs and a DCR of 30% were observed in 43 patients with microsatellite‐stable colorectal cancer.^14^ INCB001158 also has been investigated in combination with gemcitabine/cisplatin in a phase I/II study in patients with advanced biliary tract cancer, demonstrating an ORR of 24% and SD in 42% of patients.^15^ Thus, there is some evidence that INCB001158 is active when combined with other agents.

In immunohistochemistry studies of human tumor tissue microarrays, arginase‐1‐positive infiltrating granulocytes were found in abundance in a wide range of tumor types.^13^ However, there was considerable variation between patients in arginase‐1 expression, with some expressing undetectable levels. Thus, it is feasible that the small number of patients in the INCB001158‐treated groups in the current study had tumors that expressed low levels of arginase‐1. Further evaluations, including the number and activation of tumor‐infiltrating immune cells, are needed to determine tumor types most likely to respond to combination treatments with arginase inhibitors such as INCB001158.

The primary objective of this study was to assess the safety and tolerability of retifanlimab in combination with INCB001158 in an initial small sample of patients, with evaluation of preliminary evidence of clinical activity a secondary objective. As such, the small sample size in each group could have resulted in insufficient patients to detect activity in these heavily pretreated patient populations. Additionally, it is hard to draw conclusions regarding activity owing to the wide variety of tumor types and patients' high levels of prior treatment, although these were typical for a study of this type.

In conclusion, the encouraging safety and antitumor activity of retifanlimab in this study support its further investigation in Japanese patients with advanced solid tumors and confirmed the RP2D of 500 mg administered Q4W to be suitable in this population. The study did not demonstrate benefit of combined treatment with retifanlimab and INCB001158. Further investigations are required to determine biomarkers that could indicate which Japanese patients are most likely to respond to INCB001158 or the retifanlimab and INCB001158 treatment combination.

## AUTHOR CONTRIBUTIONS

**Yasutoshi Kuboki:** Data curation (equal); writing – original draft (equal); writing – review and editing (equal). **Takafumi Koyama:** Data curation (equal); writing – original draft (equal); writing – review and editing (equal). **Nobuaki Matsubara:** Data curation (equal); writing – original draft (equal); writing – review and editing (equal). **Yoichi Naito:** Data curation (equal); writing – original draft (equal); writing – review and editing (equal). **Shunsuke Kondo:** Data curation (equal); writing – original draft (equal); writing – review and editing (equal). **Kenichi Harano:** Data curation (equal); writing – original draft (equal); writing – review and editing (equal). **Kan Yonemori:** Data curation (equal); writing – original draft (equal); writing – review and editing (equal). **Kiyotaka Yoh:** Data curation (equal); writing – original draft (equal); writing – review and editing (equal). **Yuan Gu:** Formal analysis (equal); writing – original draft (equal); writing – review and editing (equal). **Tetsuya Mita:** Formal analysis (equal); writing – original draft (equal); writing – review and editing (equal). **Xuejun Chen:** Formal analysis (equal); writing – original draft (equal); writing – review and editing (equal). **Eiji Ueda:** Formal analysis (equal); writing – original draft (equal); writing – review and editing (equal). **Noboru Yamamoto:** Data curation (equal); writing – original draft (equal); writing – review and editing (equal). **Toshihiko Doi:** Data curation (equal); writing – original draft (equal); writing – review and editing (equal). **Toshio Shimizu:** Data curation (equal); writing – original draft (equal); writing – review and editing (equal).

## FUNDING INFORMATION

This study was funded by Incyte Corporation (Wilmington, DE, USA).

## CONFLICT OF INTEREST STATEMENT

YK reports institution grants or contracts from AbbVie, Astellas, AstraZeneca, Boehringer Ingelheim, Chugai, Daiichi‐Sankyo, Eli Lilly & Company, Genmab, GlaxoSmithKline, Incyte Corporation, Taiho Pharmaceutical, and Takeda; consulting fees from Amgen, Boehringer Ingelheim, Taiho Pharmaceutical, and Takeda; payment or honoraria from Bristol Myers Squibb, Eli Lilly & Company, Ono Pharmaceutical, and Taiho Pharmaceutical. TK reports grants from Daiichi‐Sankyo, Eli Lilly & Company, Novartis, and PACT Pharma; personal fees from Chugai and Sysmex outside the submitted work. NM reports institution research funding from AbbVie, Astellas, AstraZeneca, Amgen, Bayer, Chugai, Eisai, Eli Lilly & Company, Janssen Pharma, MSD, Novartis, PRA Health Science, Pfizer, Roche, Seagen, Taiho, and Takeda; speakers' bureau for Sanofi. YN reports institution grants from the Ministry of Health, Labour and Welfare; institution grants or contracts from AbbVie, AstraZeneca, Bayer, Boehringer Ingelheim, Chugai, Daiichi‐Sankyo, Eisai, Eli Lilly & Company, Ono Pharmaceutical, Pfizer, and Taiho Pharmaceutical; payment or honoraria from AstraZeneca, Bristol Myers Squibb, Chugai, Eisai, Eli Lilly & Company, Fuji Film Toyama Chemistry, Gardant, Mundipharma, Novartis, Ono Pharmaceutical, Pfizer, Shionogi, Taiho Pharmaceutical, and Takeda. SK reports research funding from AbbVie, AstraZeneca, Boehringer Ingelheim, Chugai, Eisai, Eli Lilly & Company, Incyte Corporation, MSD, Pfizer, and Takeda; consulting fees or honorariums from Chugai, Eisai, Incyte Corporation, Takeda, and Terumo. KH reports speaker or advisory role for AstraZeneca, Chugai, Daiichi‐Sankyo, MSD, and Takeda; research funding from Daiichi‐Sankyo and Merck; non‐remunerated activities with Chugai and Merck. KY (Yonemori) reports honoraria from AstraZeneca, Boehringer Ingelheim, Chugai, Eisai, Eli Lilly & Company, Fuji Film Pharma, MSD, Ono Pharmaceutical, Pfizer, and Takeda; consultancy or advisory role for AstraZeneca, Chugai, Eisai, Genmab, Novartis, OncXerna Therapeutics, and Takeda; research support to institution from AstraZeneca, Boehringer Ingelheim, Chugai, Daiichi‐Sankyo, Eisai, Eli Lilly & Company, Genmab, Haihe Biopharma, Kyowa Hakko Kirin, MSD, Nippon Kayaku, Novartis, Ono Pharmaceutical, Pfizer, Sanofi, Seagen, Taiho Pharmaceutical, and Takeda. KY (Yoh) reports grants and personal fees from AstraZeneca, Boehringer Ingelheim, Chugai Pharma, Daiichi‐Sankyo, Eli Lilly & Company, Taiho Pharmaceutical, and Takeda; personal fees from Bristol Myers Squibb, Janssen, Kyowa Kirin, and Novartis; grants from AbbVie, MSD, and Pfizer, outside the submitted work. YG and XC report employment and stock ownership with Incyte Corporation; TM and EU report employment and stock ownership with Incyte Biosciences Japan GK. NY reports research grants from AbbVie, Astellas, AstraZeneca, Bayer, Bristol Myers Squibb, Boehringer Ingelheim, Carna Biosciences, Chiome Bioscience, Chugai, Daiichi‐Sankyo, Eisai, Eli Lilly & Company, Genmab, GlaxoSmithKline, Janssen, Kyowa Hakko Kirin, Merck, MSD, Novartis, Ono Pharmaceutical, Otsuka, Pfizer, Shionogi, Sumitomo Dainippon, Taiho Pharmaceutical, and Takeda; advisory role for Boehringer Ingelheim, Chugai, Cimic, Eisai, Otsuka, and Takeda; speakers' bureau for AstraZeneca, Chugai, Daiichi‐Sankyo, Eisai, Eli Lilly & Company, Ono Pharmaceutical, and Sysmex. TD reports institution grants or contracts from AbbVie, Boehringer Ingelheim, Bristol Myers Squibb, Chugai Pharma, Daiichi‐Sankyo, Eisai, IQVIA, Janssen Pharma, Lilly, Merck Biopharma, MSD, Novartis, Pfizer, Sumitomo Dainippon, and Taiho; personal consulting fees from AbbVie, Bayer, Chugai Pharma, Kaken Pharma, Kyowa Kirin, Otsuka Pharma, PRA Health Science, Rakuten Medical, Shionogi, Sumitomo Dainippon, Taiho, and Takeda; personal payment or honoraria from AstraZeneca, Bristol Myers Squibb, Daiichi‐Sankyo, Ono Pharma, and Rakuten Medical; data safety monitoring board or advisory board for AbbVie, Amgen, Astellas, Bayer, Boehringer Ingelheim, Daiichi‐Sankyo, Janssen Pharma, MSD, and Novartis. TS reports research grants from 3D‐Medicine, AbbVie, Astellas, AstraZeneca, Bristol Myers Squibb, Chordia Therapeutics, Daiichi‐Sankyo, Eisai, Eli Lilly & Company, Incyte Corporation, LOXO Oncology, Novartis, Pfizer, PharmaMar, Symbio Pharmaceuticals, and Takeda Oncology, and 3D‐Medicine; advisory role and consulting fees from AbbVie, Chordia Therapeutics, and Daiichi‐Sankyo; speakers' bureau for Chugai, Eli Lilly & Company, and MSD.

## ETHICS STATEMENT

The study was performed in accordance with the ethical principles of the Declaration of Helsinki, local regulatory requirements, and each center's institutional review board and/or independent ethics committee. Written informed consent was obtained from each patient prior to participation. The clinical trial was registered at clinicaltrials.gov, registration number NCT03910530, and clinicaltrials.jp, registration number JapicCTI‐194882.

## Supporting information


Appendix S1.

## Data Availability

Access to individual patient‐level data is not available for this study.
