# Supplementary material for: PD‐1 inhibition with retifanlimab and/or arginase inhibition with INCB001158 in Japanese patients with solid tumors: A phase I study
Source: Cancer Med. 2024 Apr 23;13(8):e6980. doi: 10.1002/cam4.6980 (PMC11036078; doi:10.1002/cam4.6980)

**SUPPORTING INFORMATION**

**PD-1 inhibition with retifanlimab and/or arginase inhibition with INCB001158 in Japanese patients with solid tumors: A phase I study**

Yasutoshi Kuboki^1^ | Takafumi Koyama^2^ | Nobuaki Matsubara^3^ | Yoichi Naito^4^ | Shunsuke Kondo^2^ | Kenichi Harano^1^ | Kan Yonemori^2^ | Kiyotaka Yoh^5^ | Yuan Gu^6^ | Tetsuya Mita^7^ | Xuejun Chen^6^ | Eiji Ueda^7^ | Noboru Yamamoto^2^ | Toshihiko Doi^1^ | Toshio Shimizu^2,8^

^1^Department of Experimental Therapeutics, National Cancer Center Hospital East, Kashiwa, Japan

^2^Department of Experimental Therapeutics, National Cancer Center Hospital, Tokyo, Japan

^3^Department of Breast and Medical Oncology, National Cancer Center Hospital East, Kashiwa, Japan

^4^Department of General Internal Medicine, National Cancer Center Hospital East, Kashiwa, Japan

^5^Department of Thoracic Oncology, National Cancer Center Hospital East, Kashiwa, Japan

^6^Incyte Corporation, Wilmington, DE, USA

^7^Incyte Biosciences Japan G.K., Tokyo, Japan

^8^Department of Medical Oncology/Cancer Center, Wakayama Medical University Hospital, Wakayama Medical University Graduate School of Medicine, Wakayama, Japan

**SUPPLEMENTAL TABLES**

**TABLE S1** Definitions of dose-limiting toxicities

| **General** | |
| --- | --- |
| - Any death not clearly due to the underlying disease or extraneous causes. - Any grade 2 toxicity that (in the opinion of the investigator) is potentially life-threatening and not able to be controlled with standard measures, such as corticosteroids. - Drug-related AE of any grade reported during the DLT evaluation period leading to a continuous drug interruption, and preventing a patient from receiving ≥75% of the planned cohort-specified doses of study treatment. | |
| **Hematologic toxicity** | |
| - Grade 4 thrombocytopenia or grade ≥3 thrombocytopenia with clinically significant bleeding (requires hospitalization, transfusion of blood products, or other urgent medical intervention). - Grade ≥4 neutropenia lasting >5 days. - Febrile neutropenia. - Grade 4 anemia not explained by underlying disease or unrelated illnesses, such as hemolysis. - Thrombocytopenia requiring platelet transfusion, or anemia requiring red blood cell transfusion. | |
| **Nonhematologic toxicity** | |
| - Any grade ≥3 nonhematologic toxicity EXCEPT for the following:   - Transient (≤72 hours) abnormal laboratory values not requiring management, such as amylase.   - Grade 3 nausea/vomiting or diarrhea <72 hours with adequate antiemetic and other supportive care.   - Grade 3 fatigue <1 week.   - Asymptomatic lipid profile or blood glucose changes.   - Alopecia. - Events meeting Hy's Law criteria (defined as an increase in AST or ALT >3 × ULN and total bilirubin >2 × ULN, with no other reason to explain the combination of increases). | |
| **Immune-related toxicity** | |
| - Grade 3 irAEs not improving to baseline or at least grade 1 in ≤5 days with appropriate care or with corticosteroid therapy will be considered a DLT. Exception: grade 3 rash in the absence of desquamation, no mucosal involvement, no systemic steroids required, and resolves to grade 1 by the next scheduled dose of retifanlimab or 14 days, whichever is longer. - Grade 4 irAEs will be considered a DLT regardless of duration. | |
| **Urea cycle inhibition event** | **Definition** |
| Symptomatic hyperammonemia | Plasma ammonia ≥2 × ULN and ≥2 × baseline, with symptoms. |
| Orotic acid elevation accompanied by hyperammonemia | Urinary orotic acid elevation that meets either of the following criteria in 2 consecutive measurements:   - Fasting urinary orotic acid >10 × ULN (repeated measurements)   ***OR***   - Any urinary orotic acid >40 × ULN (repeated measurements) accompanied by plasma ammonia elevation ≥2 × ULN and ≥2 × baseline, at the same time as one of the 2 orotic acid measurements. |

Abbreviations: AE, adverse event; ALT, alanine aminotransferase; AST, aspartate aminotransferase; DLT, dose-limiting toxicity; irAE, immune-related adverse event; ULN, upper limit of normal.

**TABLE S2** Summary of retifanlimab pharmacokinetic parameters after first dose and steady-state in Japanese patients (POD1UM-104), and comparison with Western population (POD1UM-101^a^)

| **Study** | **n** | **First dose** | | | | | | | | | **Steady-state** |
| --- | --- | --- | --- | --- | --- | --- | --- | --- | --- | --- | --- |
|  |  | **C_max_ (mg/L)** | **C_tau_ (mg/L)** | **t_max_ (h)** | **AUC_tau_ (mg•day/L)** | **AUC_0-∞_(mg•day/L)** | **t_½_ (days)** | **CL (L/day)** | **V_z_ (L)** | **C_tau_**^b^ **(mg/L)** | |
| POD1UM-104 | 6 | 209  ± 17.7 (209) | 34.8  ± 9.31 (33.9) | 1.9 (1.1, 7.1) | 1930  ± 301 (1910) | 3080  ± 858 (2990) | 21.7  ± 5.29 (21.2) | 0.172  ± 0.0428 (0.167) | 5.13  ± 0.383 (5.12) | 83.9  ± 26.2 (81.1) | |
| POD1UM-101 (500 mg Q4W expansion cohort) | 40 | 168  ± 51.6 (159) | — | — | 1370  ± 437  (1300) | 1940  ± 795  (1800) | — | 0.302  ± 0.132 (0.279) | 6.34  ± 2.49 (5.90) | 58.7  ± 26.8 (52.9) | |

*Note:* Values are mean ± standard deviation (geometric mean) except t_max_, which is median (min, max).
Abbreviations: AUC_0-∞_, area under the serum concentration–time curve from time 0 to infinity; AUC_tau_, area under the serum concentration–time curve from time 0 to end of dosing interval; CL, clearance; C_max_, maximum observed serum concentration; C_tau_, serum concentration observed at end of dosing interval; Q4W, every 4 weeks; t_½_, apparent terminal-phase disposition half-life; t_max_, time to maximum observed serum concentration; V_z_, volume of distribution.

^a^Study POD1UM-101 (NCT03059823).

^b^Steady-state C_tau_ was determined in 3 patients in study POD1UM-104.

**TABLE S3** Summary of retifanlimab pharmacokinetic parameters after first dose in combination with INCB001158

| **n** | **C_max_ (mg/L)** | **C_tau_ (mg/L)** | **t_max_ (h)** | **AUC_tau_ (mg•day/L)** |
| --- | --- | --- | --- | --- |
| 3 | 187 ± 0.577 (187) | 19.9 ± 7.22 (19.0) | 0.82 (0.78, 6.0) | 2330 ± 568 (2280) |

*Note:* Values are mean ± standard deviation (geometric mean) except t_max_, which is median (min, max).
Abbreviations: AUC_tau_, area under the serum concentration–time curve from time 0 to end of dosing interval; C_max_, maximum observed serum concentration; C_taut_, serum concentration observed at end of dosing interval; t_max_, time to maximum observed serum concentration.

**TABLE S4** Summary of INCB001158 pharmacokinetics at first dose and steady-state

| **First dose** | | | | | | | | | |
| --- | --- | --- | --- | --- | --- | --- | --- | --- | --- |
| **Cohort** | **Dose** | **n** | **C_max_ (ng/mL)** | **t_max_ (h)** | **AUC_tau_ (ng•h/mL)** | **AUC_0-∞_ (ng•h/mL)** | **t_½_ (h)** | **CL/F (L/h)** | **V_z_/F (L)** |
| INCB001158 monotherapy | 75 mg BID | 3 | 1280 ± 261 (1270) | 3.9 (3.9, 4.0) | 10,400 ± 1920 (10,300) | 15,800 ± 5950 (15,100) | 5.95 ± 2.98 (5.51) | 5.22 ± 1.96 (4.98) | 40.2 ± 8.04 (39.6) |
| INCB001158 monotherapy | 100 mg BID | 3 | 1750 ± 239 (1740) | 3.9 (2.0, 3.9) | 13,900 ± 3590 (13,700) | 19100 ± 8300 (17,900) | 4.87 ± 2.11 (4.55) | 5.90 ± 2.31 (5.57) | 36.7 ± 4.15 (36.6) |
| INCB001158 + retifanlimab combination^a^ | 100 mg BID | 6 | 1790 ± 336 (1770) | 5.9 (3.8, 6.0) | 15,000 ± 3510 (14,700) | 30,400, 19,900 | 5.92, 4.43 | 3.29, 5.01 | 28.1, 32.0 |

| **Steady-state** | | | | | | | | | |
| --- | --- | --- | --- | --- | --- | --- | --- | --- | --- |
| **Cohort** | **Dose** | **n** | **C_max_ (ng/mL)** | **t_max_ (h)** | **AUC_tau_ (ng•h/mL)** | **C_tau_ (ng•h/mL)** | **t_½_ (h)** | **CL/F (L/h)** | **V_z_/F (L)** |
| INCB001158 monotherapy | 75 mg BID | 3 | 2140 ± 546 (2100) | 3.9 (3.8, 4.0) | 19,300 ± 4370 (18,900) | 940 ± 269 (911) | 5.74 ± 1.22 (5.66) | 4.05 ± 1.04 (3.96) | 33.0 ± 7.37 (32.4) |
| INCB001158 monotherapy^†^ | 100 mg BID | 3 | 2710 ± 361 (2690) | 3.8 (2.0, 5.8) | 23,700 ± 6150 (23,200) | 1180 ± 421 (1130) | 6.25, 5.50 | 4.39 ± 0.991 (4.31) | 44.9, 39.3 |
| INCB001158 + retifanlimab combination | 100 mg BID | 5 | 2570 ± 555 (2510) | 4.0 (3.8, 6.0) | 23,500 ± 5960 (22,800) | 1340 ± 412 (1280) | 8.98 ± 5.18 (8.11) | 4.56 ± 1.54 (4.39) | 51.6 ± 30.0 (46.6) |

*Note:* Values are mean ± standard deviation (geometric mean) except t_max_, which is median (min, max).
Abbreviations: AUC_0-∞_, area under the plasma concentration–time curve from time 0 to infinity; AUC_tau_, area under the plasma concentration–time curve from time 0 to end of dosing interval; BID, twice daily; CL/F, apparent oral dose clearance; C_max_, maximum observed plasma concentration; C_tau_, plasma concentration observed at end of dosing interval; t_½_, apparent terminal-phase disposition half-life; t_max_, time to maximum observed plasma concentration; V_z_/F, apparent oral dose volume of distribution.

*AUC_0-∞_, t_½_, CL/F, and V_z_/F were calculated in n = 2.
^†^t_½_ and V_z_/F were calculated in n = 2.

**SUPPLEMENTAL FIGURES**

**FIGURE S1** Retifanlimab monotherapy (A) serum concentration after first dose and (B) serum trough concentration from cycle 1 to cycle 7. Data presented as mean ± standard error


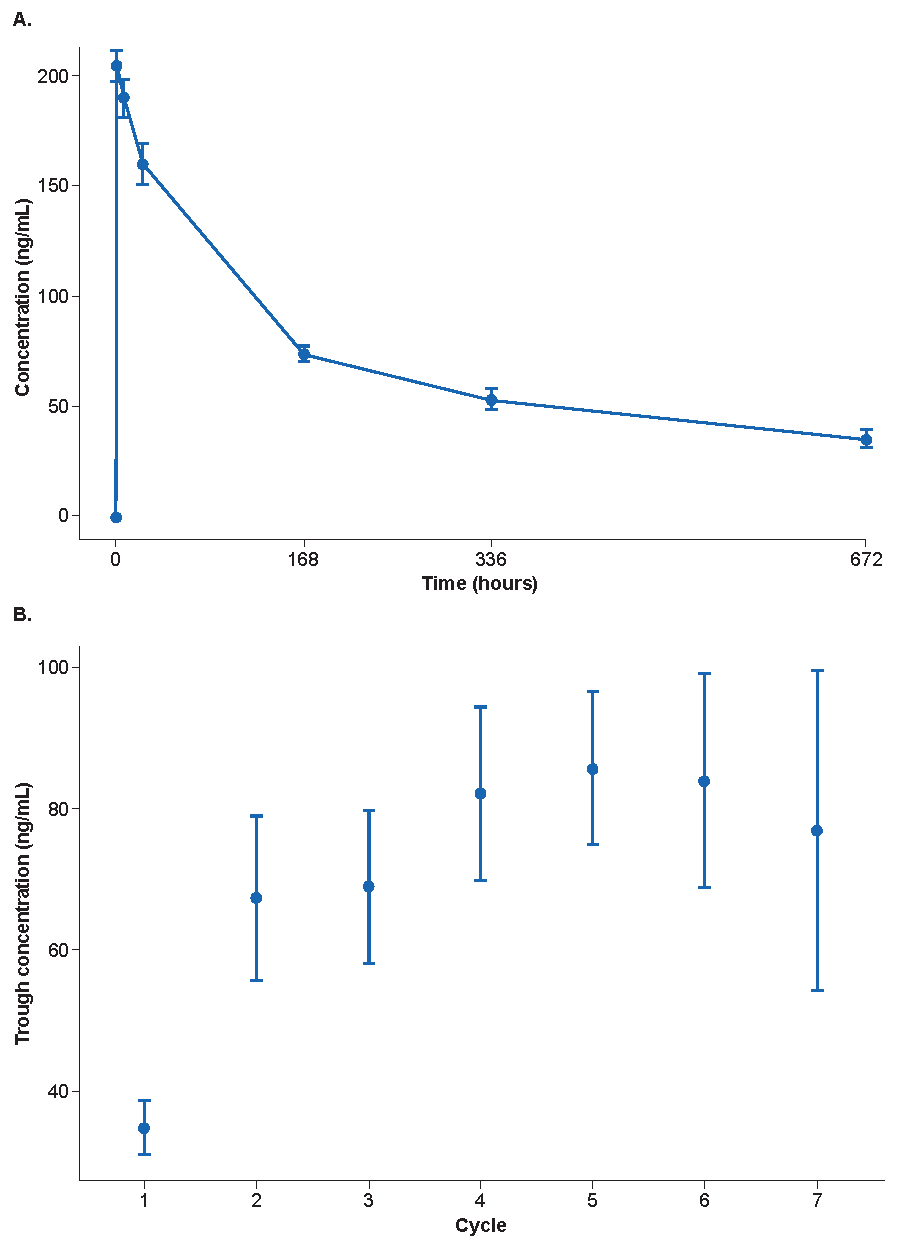


**FIGURE S2** INCB001158 plasma concentration (A) as monotherapy and (B) in combination with retifanlimab. Data presented as mean ± standard error


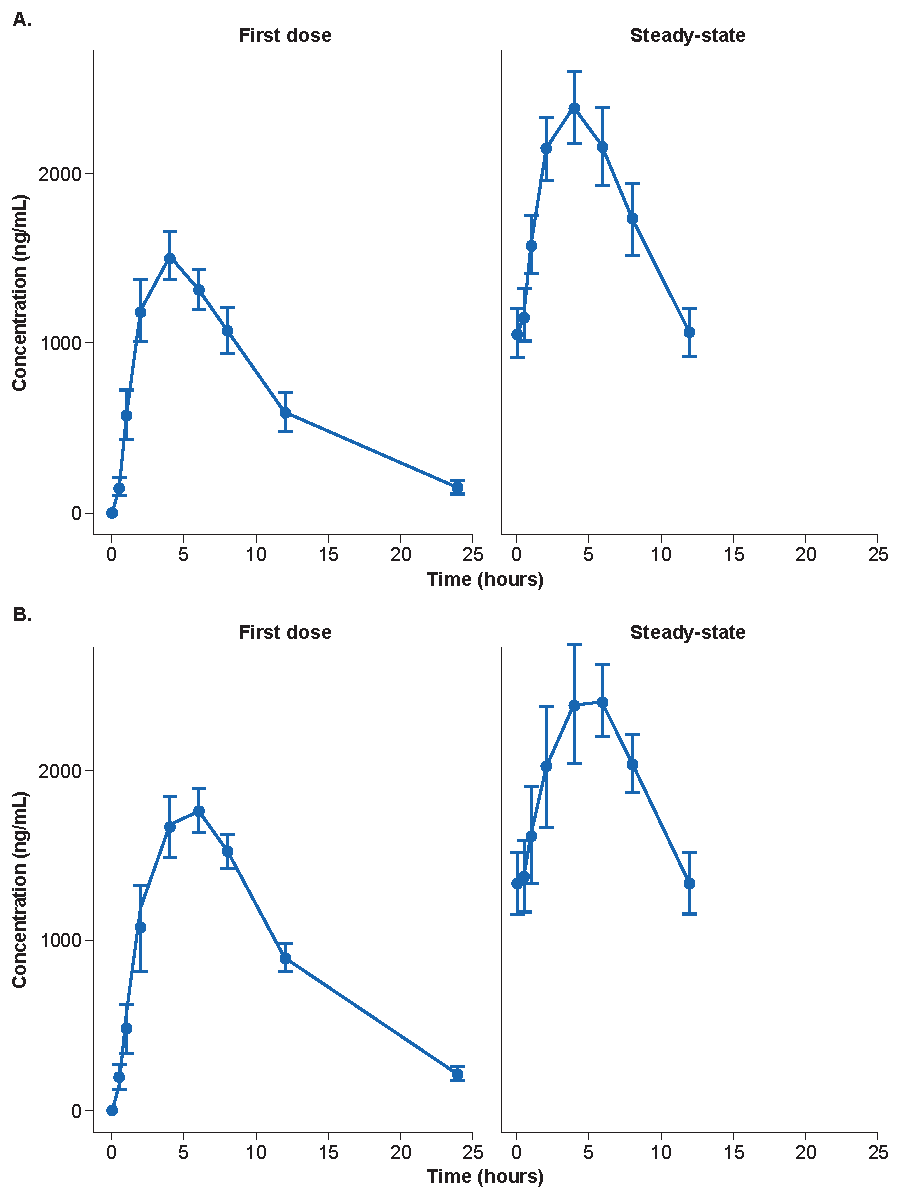


**FIGURE S3** Percentage change from baseline in (A) plasma arginine, (B) urine orotic acid, and (C) plasma ammonia among patients receiving INCB001158 alone or in combination with retifanlimab. C, cycle; D, day; EOT, end of treatment; pre, pre-dose; post, post-dose


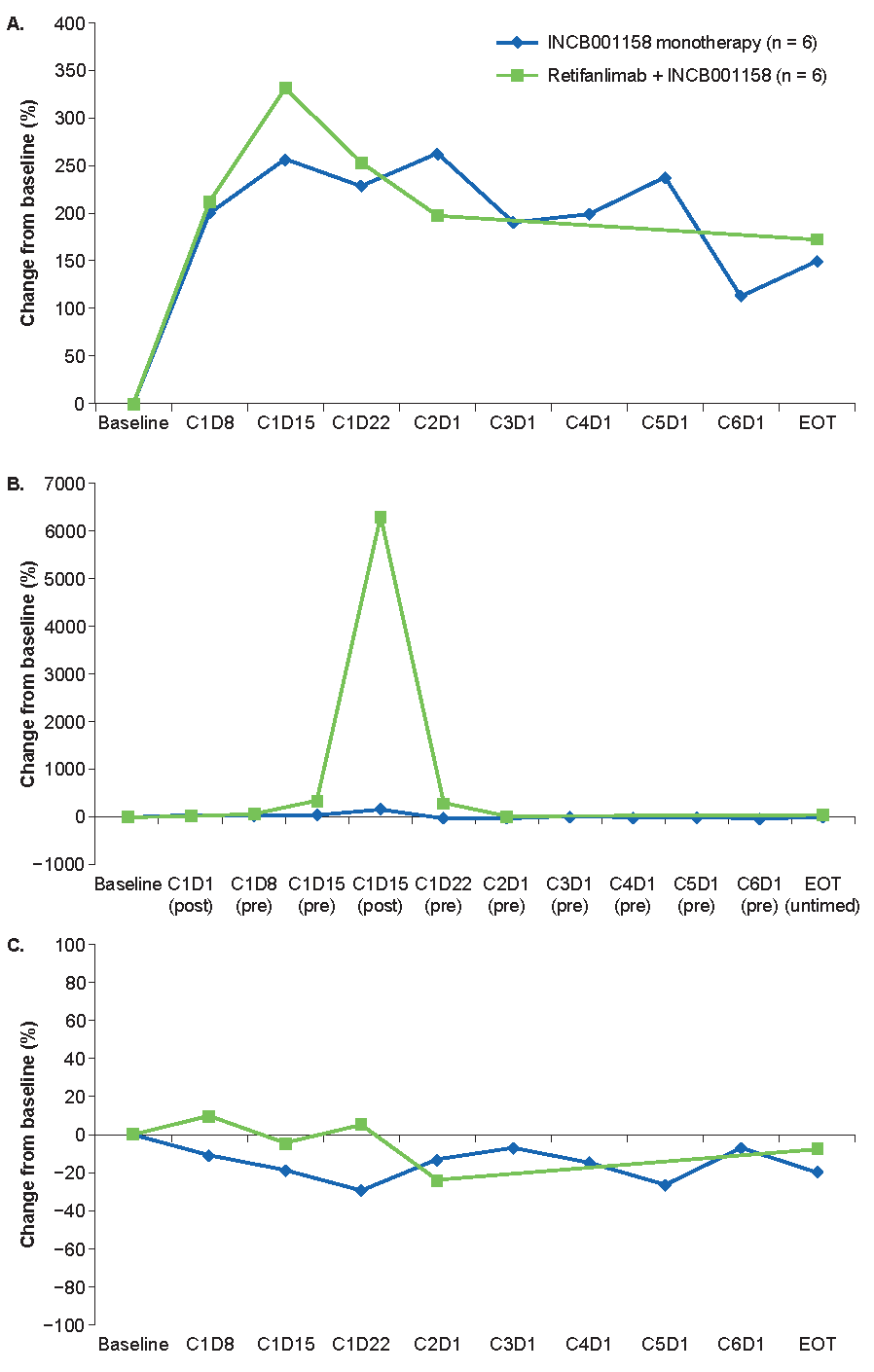

Supplement: Supplementary file 1 — Appendix S1. [file CAM4-13-e6980-s001.docx]
